# Chicago Gynecological Society

**Published:** 1885-05

**Authors:** 

**Affiliations:** 2330 Indiana Avenue


### SOCIETY PROCEEDINGS.

---

TRANSACTIONS OF THE CHICAGO GYNECOLOGICAL SOCIETY.—*Friday Evening, March 20, 1885. Regular Meeting.* I.—THE INFLUENCE OF CIMICIFUGA RACEMOSA UPON PARTURITION, *by* J. SUYDAM KNOX, M. D. II.—SOME REMARKS ON THE VALUE OF PERMANGANATE OF POTASH IN AMENORRHŒA—*Inaugural Thesis, by* EDMUND J. DOERING, M. D. III.—REPORT OF A CASE OF HYSTERECTOMY FOR INTRA-MURAL MYOMA, *by Professor* E. C. DUDLEY, M. D.

*The President,* DR. P. H. MERRIMAN, in the chair.

I. Professor J. Suydam Knox read a paper entitled, "The Influence of Cimicifuga Racemosa upon Parturition."

After a *résumé* of the medical history of the drug, Professor Knox gave the results of his clinical observations in one hundred and fifty cases of labor,—fifty-seven primiparæ, ninety-three multiparæ,—in which black cohosh had been exhibited. The average duration of the first and second stages of labor, in normal cases, in primiparæ, was seventeen and three hours, respectively. Under the influence of black cohosh, the duration of the first and second stages of labor, in the fifty-seven cases observed, was six and one-quarter, and one and three-quarter hours, respectively. The average duration of the first and second stages, in normal cases, in multiparæ, was twelve and one hours, respectively. Under the influence of black cohosh, in the ninety-three cases observed, the average duration of the first and second stages was three hours, and twenty-seven minutes, respectively.

From these clinical observations Dr. Knox drew the following conclusions:

"1. Cimicifuga has a positive sedative effect upon the parturient woman, quieting reflex irritability. Nausea, pruritus and insomnia, so common in the last six week of pregnancy, are always rendered less distressing, and often disappear under its administration.

"2. Cimicifuga has a positive anti-spasmodic effect upon the parturient woman. The neuralgic cramps and irregular pains of the first stage of labor are ameliorated, and often altogether abolished. In fact, during the first indiscriminate use of the drug in all cases, I had the mortification, with a few women, of terminating the labor so precipitately, and without prodromic symptoms, as to be unable to reach the bedside before the birth.

"3. Cimicifuga relaxes uterine muscular fibre, and the soft parts of the parturient canal, by controlling muscular irritability, thus facilitating labor and diminishing risks of laceration.

"4. Cimicifuga increases the energy and rhythm of the pains in the second stage of labor.

"5. It is my belief that cimicifuga, like ergot, maintains a better contraction of the uterus after delivery.

"It is my habit, however, to administer fifteen to thirty minims of the fluid extract of ergot after the birth of the fœtal head, and I have had but few opportunities of testing this effect of the cohosh.

"My method of administration has been to give fifteen minims fluid extract of cimicifuga in compound syrup of sarsaparilla each night for four weeks before the expected confinement.

"One fluid ounce of the fluid extract of cimicifuga to three fluid ounces of compound sarsaparilla,—dose, one teaspoonful,—makes just the required quantity."

*Discussion.*—Dr. Philip Adolphus had employed the cohosh in the manner indicated by Professor Knox, in one case, with negative results.

Dr. Edward Warren Sawyer thought the results obtained by Professor Knox were astonishing. He thought there could be no doubt that the drug had the physiological action to which allusion has been made. He would, at once, act upon the suggestion in his private practice.

Professor W. W. Jaggard thought that if the influence upon parturition, so clearly sketched by Professor Knox, was capable of demonstration, he could agree with Dr. Sawyer's panegyric. Professor Knox's carefully prepared paper was worthy of study and investigation. Dr. H. Webster Jones, formerly a prominent obstetrician in Chicago, had advanced similar views, with reference to the physiological action of black cohosh, in a paper published in the *Transactions of the Illinois State Medical Society*, a few years since. Dr. Jones's advocacy of the drug as an oxytocic was well known to every practitioner in the city.

Professor Jaggard desired to call the attention of the Fellows to the following subjects in Professor Knox's paper:

I. Professor Knox had stated that the average duration of the first stage of labor in primiparæ and multiparæ was, respectively, seventeen and twelve hours; under the influence of black cohosh, the duration of this stage was abbreviated to six and one-quarter, and three hours, respectively. It was a matter of extreme difficulty to define the limits of the duration of the first stage of labor with such mathematical accuracy. The "personal equation" assumes great importance as a possible source of error. The subjective signs and the results of physical exploration are not always sufficient to justify the diagnosis of the commencement of labor. Thus, Dr. R. Lumpe

(Archiv. für Gynaekologie, 1883, Bd. XXI. Hft. I, p. 29) concludes, from the observation of several hundred primiparæ, that the cervical canal begins to dilate from eight to fourteen days before the expulsion of the child. Other observers assign a period of much briefer duration than the typical seventeen hours of Spiegelberg, to which Dr. Knox alluded.

Apart from the difficulty in the determination of the commencement of the first stage, the duration is capable of infinite individual variation.

II. One hundred and fifty cases were insufficient to warrant such positive deductions upon an intricate therapeutical problem. In every one of the cases cited by Dr. Knox, it was clearly indeterminate whether or no the effect was *post hoc* or *propter hoc*.

Black cohosh had been employed, on an extensive scale, in large lying-in hospitals in Germany. Every condition for accurate clinical observation had been supplied. Such conditions were: competent observers, numerous cases, under absolute control for a sufficient period of time, chemical purity of the drug, and an approximately perfect system of keeping records. Up to the present time, results had been of a purely negative character. It was true that Schatz had reported favorably as to the action of the drug in the treatment of certain pathological conditions of uterus.

Dr. Jaggard did not wish to be understood as dogmatically condemning the drug. The evidence in favor of its action as an oxytocic, as collected from experiments upon the lower animals, or from clinical observation, was entirely insufficient to warrant the positive conclusions of the author of the paper. The subject was worthy of further investigation.

III. He thought the practice of the exhibition of ergot, before the completion of the second stage of labor, was reprehensible. It was in conflict with the obstetrical principles of the day, as deduced from clinical experience, and the nature of the case. This remark was applicable exclusively to physiological labors.

Professor Charles Warrington Earle had used black cohosh, at the suggestion of Dr. Jones and Professor Knox, in a variety of cases, with negative results. He had about the same number of precipitate labors as occurred in his practice prior to the introduction of the drug. It was possible that he had not employed doses of sufficient size, nor for a sufficient length of time before labor.

Dr. Henry T. Byford wished to enter a protest against all methods of rendering the process of labor shorter. Quick labors were wrong labors, as a rule.

Dr. George M. Chamberlain had no experience with black cohosh, but he was opposed to the exhibition of ergot before the expulsion of the child in physiological cases.

Professor Knox closed the discussion.

In reply to Professor Jaggard, he said that the results of his clinical experience with black cohosh were of such a convincing character, that he would continue the exhibition of the drug in the future. In regard to the exhibition of ergot, before the expulsion of the child, he did it to save time. The drug was not absorbed until twenty minutes after exhibition, and long before the expiration of that time the child was born. He could not spare the twenty minutes, required to secure retraction of the uterus, after delivery of child and placenta. In reply to Dr. H. T. Byford, he said that, at the present day, there were no physiological labors. Women were not Eves. By the judicious use of a drug, like black cohosh, labor was made to resemble the ideal, physiological process, as still observed among savages.

II. The Secretary then read the inaugural thesis of Dr. Edmund J. Doering M. D., (Chicago Medical College, 1874), entitled, "Some Remarks on the Value of Permanganate of Potash in Amenorrhœa."

After a brief description of the physical and chemical character of the drug, Dr. Doering discussed its physiological action. Bartholow, who has great faith in the drug, claims that although it parts with its oxygen with great readiness, the readiness is *not* sufficiently great to prevent the distribution of the gas into the blood. "His opponents deny this, and argue that the organic matter contained in the stomach and mucous membranes is sufficient to appropriate the oxygen of the salt and thus prevent its entrance into the circulation."

Professor N. Gray Bartlett, a Chicago chemist, gives the following opinion:

"From the readiness with which the permanganate of potassium is decomposed by organic compounds, it would seem to be ineligible for universal use. When so administered, it is immediately brought into contact, in the stomach, with a relatively large amount of organic matter and necessarily is very rapidly destroyed, the manganese of the permanganate separating, in all probability, in the form of the hydrated manganese dioxide. The latter compound is an active oxidizing agent, and is possibly capable of exercising in the economy the oxidizing function, which has been ascribed to the permanganate of potassium. It would seem rational, therefore, anticipating the change which follows the administration of the permanganate, to substitute the hydrated dioxide of manganese, which can readily be prepared in a state of purity for medical use."

Whatever view may be adopted as to the chemical change which the permanganate undergoes in the human economy, the main question is as to its therapeutic value.

Professor T. Gaillard Thomas, M. D., in his address to the New York State Medical Association, expresses faith in the value of the drug as an emmenagogue. Dr. Ringer and Dr. Murrell recommend the remedy. Dr. Doering had given the drug a careful trial in thirty cases of amenorrhœa, depending upon anæmia and general atony of the sexual apparatus. In about half the cases the observations were unsatisfactory, from various causes, *i. e.*, inattention to the general directions, want of perseverance in taking the medicine, so that the conclusions arrived at were based upon fourteen cases, in each of which the cause of the amenorrhœa was entirely clear, the remedy carefully and continuously given, and the effect clearly observed.

The cases were tabulated.

*Conclusions.*—I. Permanganate of potash in doses of from two to four grains is an efficient emmenagogue, if administered for a period of not less than two weeks.

II. Its administration in doses large enough to be effective is accompanied by severe pain, which frequently necessitates discontinuance of the remedy, and hence impairs its value as an emmenagogue.

III. The most efficient method of administering the drug is in capsules, taken midway between meals, and followed by draughts of some pure mineral water, like silurian.

Dr. Doering was elected Fellow of the Society.

Professor E. C. Dudley gave a very brief report of a case of hysterectomy for intra-mural myoma; the uterus, ovaries and tubes of which were exhibited at the February meeting of the Society.

The patient died of general peritonitis on the sixth day after operation.

The next meeting of the Society will be held on Friday evening, April 17th, 1885, at the Palmer House.

*Programme.*—Remarks upon chronic peri-uterine abscess, by Professor Christian Fenger, M. D., with the exhibition of specimens.

II. Remarks upon a Teratom, by W. W. Jaggard, M. D., with presentation of specimen.

III. A paper by Henry T. Byford, M. D.; subject to be announced.

W. W. JAGGARD, M. D., *Editor.*

2330 Indiana Avenue.

March 21st, 1885.

